# Editorial: Liquid-biopsy-guided biomarker and drug discovery

**DOI:** 10.3389/fphar.2024.1454960

**Published:** 2024-07-16

**Authors:** Olesya A. Kharenko, Edward van der Horst

**Affiliations:** ^1^ Syantra Inc, Calgary, AB, Canada; ^2^ Sensei Biotherapeutics, Gaithersburg, MD, United States

**Keywords:** liquid bioposy, drug discovery, breast cancer, circulating tumor cells, NK cells, immunotherapy, young-onset breast cancer, cancer detection

Early detection and diagnosis of cancer can significantly improve mortality rates and treatment outcomes. Liquid biopsy is an emerging field offering a convenient, non-invasive way of detecting cancer signals from biological samples such as blood, saliva, urine, or other fluids. Several liquid biopsy tests have been developed and implemented in clinical practice in recent years, including multicancer and single-cancer detection tests. Many of these tests are based on circulating tumor cells (CTCs), circulating tumor DNA (ctDNA), circulating free DNA (cfDNA), or exosomes, which still possess several limitations. Alternative approaches have recently evolved, including signatures from tumor-educated platelets, other blood cell components, blood-based multi-biomarkers, leukobiopsy, and microRNAs or oncoRNAs. These methods, coupled with machine-learning algorithms, can offer complementary or even alternative approaches to cancer diagnostics.

Conventional cancer detection is often limited to certain populations, overlooking younger or high-risk groups. For instance, breast cancer screening is typically offered only to women over 45 years or with a family history of breast cancer. However, breast cancer cases in younger women are rising, often developing more aggressive cancers that, due to a lack of early diagnosis, are detected only at advanced stages. Moreover, conventional screening methods, such as mammography, often lack sensitivity in certain populations, including women with dense breasts, necessitating additional screening methods. Stibbards-Lyle et al., in their comprehensive review, outlined the limitations of existing cancer detection methods, leading to the continuous rise in cases of young-onset breast cancer (YOBC) and postpartum breast cancers (PPBCs). They described the unique biology behind YOBC and highlighted the necessity for more sensitive and convenient detection methods, like blood-based liquid biopsies, to include overlooked populations and cover gaps in clinical care. They discussed the potential advantages of blood-based liquid biopsies, including early detection of biologically distinct cancers, which would provide more timely interventions and possibly better outcomes for patients.

Tumor progression often leads to metastatic spread to distant sites with different biological characteristics and heterogeneity compared to the primary tumor, leading to differences in therapeutic resistance levels, making treatment difficult. Liquid biopsies offer a unique advantage over conventional tissue biopsies as they allow continuous monitoring of disease progression, providing a convenient way of tracking response to treatment.

Rios-Hoyo et al. described in their review how liquid biopsy can be utilized as a tool in colon cancer to monitor the evolution of genomic and epigenetic alterations during treatment resistance and progression. The mutational burden can differ between metastatic tumor sites, making it difficult to identify the right treatment. The authors comprehensively reviewed the complex mechanisms of primary and secondary resistance to anti-EGFR therapies, often appearing upon therapeutic pressure, leading to ongoing tumor evolution and differential mutational burdens between metastatic sites. A liquid biopsy approach can be extremely beneficial, providing the opportunity for continuous, minimally invasive re-biopsy to monitor tumor heterogeneity and the evolution of the resistance profile. This can further enhance precision medicine, allowing the proper selection of combination treatments.

With the rise of immunotherapy treatments, there is a greater unmet need to develop diagnostic methods to stratify patients who will respond to immune checkpoint inhibitors (ICIs). Ando et al. discovered plasma biomarkers as a potential tool to predict recurrence and efficacy of immunotherapy, which can be useful in clinical practice for stratifying patients, identifying those who will benefit from immunotherapy, and preventing overtreatment of non-responders.

In a related study, Li et al. developed a biomarker-based signature aiming to predict the response to immunotherapy in colorectal cancer patients. Interestingly, the authors considered the importance of the innate immune response and incorporated natural killer (NK) cell regulation to generate predictive NK-related scores to evaluate treatment response. Their prognostic model, based on NK-related genes, was able to stratify responders versus non-responders to immunotherapy. Additionally, the authors validated SLC2A3 as a potential therapeutic target and biomarker for colorectal cancer.

Liquid biopsy not only offers the convenience of monitoring treatment response in real-time but also the possibility of detecting adverse events (AEs) associated with treatments. Kashiwada et al. described a novel method of identifying immune-related adverse events associated with immune checkpoint inhibitor treatments in gastric and non-small cell lung (NSCLC) cancers. This approach allows the selection of immune checkpoint inhibitors targeting durable anti-tumor responses without the toxicity associated with such therapies. This application of liquid biopsy serves as a non-invasive method to fine-tune the balance between treatment efficacy and adverse events, helping to stratify patients.

In conclusion, this Research Topic covers a wide range of liquid biopsy applications as an evolving approach in cancer diagnostics and precision medicine, significantly improving patient care and outcomes. The contributing authors, through original research and review articles, shed light on this exciting new field as a useful tool addressing unmet needs in current diagnostic and cancer treatment care.

